# Serological evidence of Ebola virus exposure in dogs from affected communities in Liberia: A preliminary report

**DOI:** 10.1371/journal.pntd.0007614

**Published:** 2019-07-22

**Authors:** Brien K. Haun, Varney Kamara, Abigail S. Dweh, Kianalei Garalde-Machida, Saymajunkon S. E. Forkay, Melissa Takaaze, Madhuri Namekar, Teri Ann S. Wong, Ayesha E. R. Bell-Gam Woto, Peter Humphreys, Ophelia I. Weeks, Mosoka P. Fallah, John M. Berestecky, Vivek R. Nerurkar, Axel T. Lehrer

**Affiliations:** 1 Department of Cell and Molecular Biology, John A. Burns School of Medicine, University of Hawaii, Honolulu, Hawaii, United States of America; 2 Department of Tropical Medicine, Medical Microbiology and Pharmacology, John A. Burns School of Medicine, University of Hawaii, Honolulu, Hawaii, United States of America; 3 Department of Biological Sciences, Medical Science, TJR Faulkner College of Science and Technology, University of Liberia, Fendall, Liberia; 4 Leon Quist Ledlum Central Veterinary Diagnostic Laboratory, Ministry of Agriculture, Republic of Liberia, Fendall, Liberia; 5 Kapiolani Community College, University of Hawaii, Honolulu, Hawaii, United States of America; 6 National Public Health Institute of Liberia, Monrovia, Republic of Liberia; University of California, Los Angeles, UNITED STATES

## Abstract

Filoviruses such as Ebola virus (EBOV) cause outbreaks of viral hemorrhagic fevers for which no FDA-approved vaccines or drugs are available. The 2014–2016 EBOV outbreak in West Africa infected approximately 30,000 people, killing more than 11,000 and affecting thousands more in areas still suffering from the effects of civil wars. Sierra Leone and Liberia reported EBOV cases in every county demonstrating the efficient spread of this highly contagious virus in the well-connected societies of West Africa. In communities, canines are often in contact with people while scavenging for food, which may include sickly bush animals or, as reported from the outbreak, EBOV infected human bodies and excrement. Therefore, dogs may serve as sentinel animals for seroprevalence studies of emerging infectious viruses. Further, due to their proximity to humans, they may have important One Health implications while offering specimens, which may be easier to obtain than human serum samples. Previous reports on detecting EBOV exposure in canines have been limited. Herein we describe a pilot project to detect IgG-responses directed against multiple filovirus and Lassa virus (LASV) antigens in dogs from EBOV affected communities in Liberia. We used a multiplex Luminex-based microsphere immunoassay (MIA) to detect dog IgG binding to recombinant filovirus antigens or LASV glycoprotein (GP) in serum from dogs that were old enough to be present during the EBOV outbreak. We identified 47 (73%) of 64 dog serum samples as potentially exposed to filoviruses and up to 100% of the dogs from some communities were found to have elevated levels of EBOV antigen-binding IgG titers. The multiplex MIA described in this study provides evidence for EBOV IgG antibodies present in dogs potentially exposed to the virus during the 2014–16 outbreak in Liberia. These data support the feasibility of canines as EBOV sentinels and provides evidence that seroprevalence studies in dogs can be conducted using suitable assays even under challenging field conditions. Further studies are warranted to collect data and to define the role canines may play in transmission or detection of emerging infectious diseases.

## Author summary

Ebola Virus (EBOV) and its related species cause hemorrhagic fevers for which there are no FDA- approved treatments. The 2014–2016 EBOV outbreak in West Africa infected over 30,000 people, killing more than 11,000. This was the largest outbreak to date and Liberia was the unfortunate epicenter. In Liberia, EBOV cases were reported in every county. While preventative and therapeutic agent developments have received much attention, prophylactic measures involving Liberian communities have seen much less attention. In Liberia, dogs may warrant surveillance as they routinely interact with animals of the forest and people within communities. Despite scavenging the excrements and even bodies of infected individuals during the outbreak, dogs reportedly remained asymptomatic for EBOV. In collaboration with the University of Liberia and the Leon Quist Ledlum Central Veterinary Diagnostic Laboratory of Liberia, our team used a multiplex Luminex-based assay to detect dog antibodies (IgG) binding recombinant filovirus antigens or LASV glycoprotein in samples from animals that were present during the EBOV outbreak. We identified several communities in which 100% of dogs showed IgG responses reactive to one or more filovirus antigens. This preliminary report establishes the feasibility of conducting EBOV seroprevalence studies in resource poor outbreak sites in Africa using modern and economical serological assay techniques.

## Introduction

Ebola virus (EBOV) was first identified in the Democratic Republic of the Congo (DRC) in 1976 [1] and has caused multiple outbreaks, resulting in 30–90% fatality rates since then [2]. EBOV, a member of the *Filoviridae* family, causes hemorrhagic fevers, and currently no FDA approved drugs or vaccines are available to prevent or treat the disease. The current EBOV outbreak in the Democratic Republic of the Congo has claimed more than 1,000 lives and is the largest EBOV outbreak in the nations’ history according to the CDC. A new EBOV strain has been implicated as the cause for the outbreak with the proposed name “Tumba” [3]. The 2013–2016 outbreak in West Africa was caused by the Makona variant of EBOV and affected nearly 30,000 people, killing more than 11,000 in West Africa. Countries such as Sierra Leone and Liberia reported EBOV cases in every county with many, including Montserrado County where Monrovia is located, counting between 501–4,000 cases [4]. The severity and global spread of this outbreak has significantly increased countermeasure efforts [5] and has focused more attention on interactions within the affected communities during these outbreaks. The World Health Organization (WHO) has developed social mobilization and community engagement campaigns for intense transmission areas [6]. These efforts highlight the broadening net of investigative measures to control and understand this disease.

Despite intense efforts, no reservoir host for the virus has yet been identified. However, epidemiologic studies have demonstrated that gorilla, chimpanzee, and duiker carcasses may in the past have been the significant sources of initial human infection [7]. The swift emergence, virulence, and disappearance of EBOV highlight the need to fully investigate the animal reservoirs that may be harboring the virus which includes both wild and domesticated animals. While vaccine candidates and antibody-based immunotherapeutics are in development to protect against or clear Ebola virus disease (EVD) [8, 9], EBOV animal reservoirs continue to remain elusive. Furthermore, the role domesticated animals may play in the community during outbreaks has remained understudied. Some evidence suggests that dogs may be infected with EBOV, however remain asymptomatic [10]. While dogs may be susceptible to infection, EBOV replication has been shown to be restricted in canine and feline cells [11], further explaining the apparent asymptomatic display of canines. This suggests that dogs are not likely to be reservoirs but may serve as sentinels as they may mount an immune response when exposed to EBOV. As dogs scavenge for food sources, they are potentially exposed to high amounts of EBOV particles present on dead animals or humans, thus they could mount a detectable IgG response against EBOV even if infection is low and unproductive.

Classically, anti-EBOV IgG has been measured using ELISA. This method was used to screen dog serum samples for anti-EBOV IgG after the 2001–2002 Gabon outbreak [10]. While ELISA is a reliable method for sero-surveillance, it is not as sensitive as the microsphere immunoassay (MIA) [12]. Additionally, ELISA is limited in that it can only detect one antigen at a time. In contrast, MIA allows for multiplexing several antigens to be detected in a single assay. Therefore, we developed a multiplex MIA to detect dog IgG reactivity to EBOV glycoprotein (GP), viral matrix protein 40 (VP40), and nucleoprotein (NP) as well as GPs of Sudan virus (SUDV), Marburg virus (MARV), and an unrelated, but endemic, Lassa virus (LASV). Using this assay we screened serum collected from domesticated dogs over 4 years of age in neighborhoods affected by the 2013–2016 EBOV outbreak in Montserrado County, Liberia. Our goal was to demonstrate that MIA-based sero-surveillance assays could be employed in resource and infrastructure poor environments in a reliable manner. The MIA-based assay described in this report provides evidence that domesticated canines may serve as unintentional sentinels for EBOV outbreaks in West Africa.

## Methods

### Sample collection

**Fig 1:**
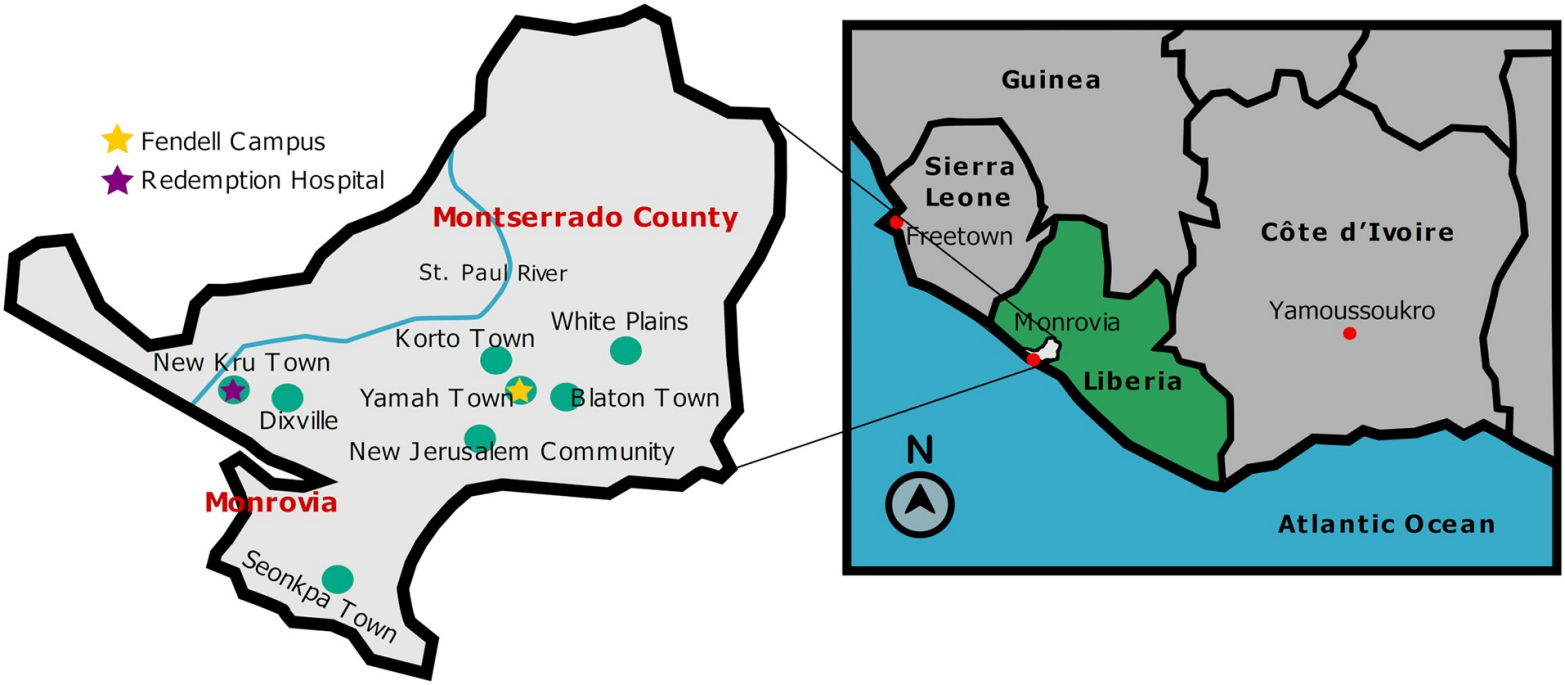
Illustrated map of communities in Montserrado County, Monrovia, Liberia. This map (not to scale) depicts communities from which dog serum samples were collected. The map on the right depicts the Southeast region of West Africa and highlights the location of Montserrado County. The image on the left is a zoomed-in illustration of Montserrado county and highlights the location of Redemption Hospital, the University of Liberia Fendall campus, and communities from which dogs were sampled, green dots. Map designed from Wikimedia Commons (https://commons.wikimedia.org/wiki/Atlas_of_Africa).

Sixty-four dog serum samples were collected during routine examinations by staff of the Ledlum Central Veterinary Diagnostic Laboratory from communities in Montserrado County, Monrovia, Liberia. Serum samples were collected between April and May 2018 from dogs within a roughly 40-mile radius around Monrovia in Montserrado County (Fig 1). All dogs were identified as being at least four years old by their owner from communities affected by the 2014–2016 EBOV outbreak. No stray dogs were used in this study. Samples were collected via cephalic vein bleeds to a volume less than 1 mL. Blood samples were allowed to clot for a minimum of 30 minutes before centrifugation at room temperature (20°C to 25°C) at 1200 x g for 10 minutes. Sera were removed from the collection tubes and transferred to cryopreservation tubes. Samples were stored under refrigeration at the Ledlum Central Veterinary Diagnostic Laboratory.

### Coupling of microspheres with recombinant antigens

The coupling of individually addressable microspheres with EBOV VP40, NP, GP, as well as SUDV GP, MARV GP, and LASV GP proteins was conducted as described previously [13, 14]. Microspheres dyed with spectrally different fluorophores were also coupled with bovine serum albumin, and PBS as controls. Internally dyed, carboxylated, magnetic microspheres (MagPlexTM-C) were obtained from Luminex Corporation (Austin, TX, USA).

### Microsphere immunoassay (MIA)

Detection of NHP and dog IgG was conducted as previously described [15]. Briefly, microspheres coupled with EBOV VP40, NP, GP, SUDV GP, MARV GP, LASV GP, and control beads coupled to PBS and BSA were combined and diluted in PBS-1% BSA at a dilution of 1/200. Fifty μL (containing approximately 1,250 beads of each type) of the microsphere suspension were added to each well of black-sided 96-well plates. Serum samples were diluted 1:1000 (NHP) or 1:200 (dog) in PBS-BSA and dog samples were serially diluted two-fold for titrations. Fifty μL of diluted serum were added to the microspheres in duplicate and incubated for 30 min on a plate shaker set at 700 rpm in the dark at room temperature. The plates were then washed twice with 200 μL of PBS-BSA using a magnetic plate separator (Millipore Corp., Billerica, MA). Fifty μL of red-phycoerythrin (R-PE) conjugated F(ab’)2 fragment rabbit anti-dog IgG (H+L) (Rockland Immunochemicals, Inc) were added at 1 μg/mL to the plates and incubated on a 96-well plate shaker for another 45 min. The plates were washed twice, as above, and microspheres were then resuspended in 100 μL of sheath fluid and analyzed on the MAGPIX Instrument (MilliporeSigma). Data acquisition detecting the median fluorescence intensity (MFI) was set to 50 beads per spectral region. Antigen-coupled beads were recognized and quantified based on their spectral signature and signal intensity, respectively.

Assay cutoff values were calculated first by taking the mean of technical duplicate values using the average MFI (indicated as a solid black line). Cut-offs were generated by determining the mean of 1/3^rd^ serum samples (22) showing the lowest MFI values plus three standard deviations as determined by Microsoft Office Excel program. Serum samples showing MFI values greater than the cutoff value were considered positive. If the cut-off value fell below the internal control BSA cutoff, the highest BSA-bead reading of 45 MFI was used (indicated by a dotted line). Graphical representation of the data was done using Prism, Graphpad Software (San Diego, CA).

## Results and discussion

Serological studies in West Africa have been conducted across a broad range of species and provide much insight into zoonotic diseases. This is the first study to report on the potential exposure of canines to filoviruses in Liberia. Furthermore, we report what appears to be a sustained level of detectable IgG up to four years after the 2014–2016 EBOV outbreak. This study is the first to implicate dogs as potential sentinels to zoonosis in West African communities.

**Fig 2:**
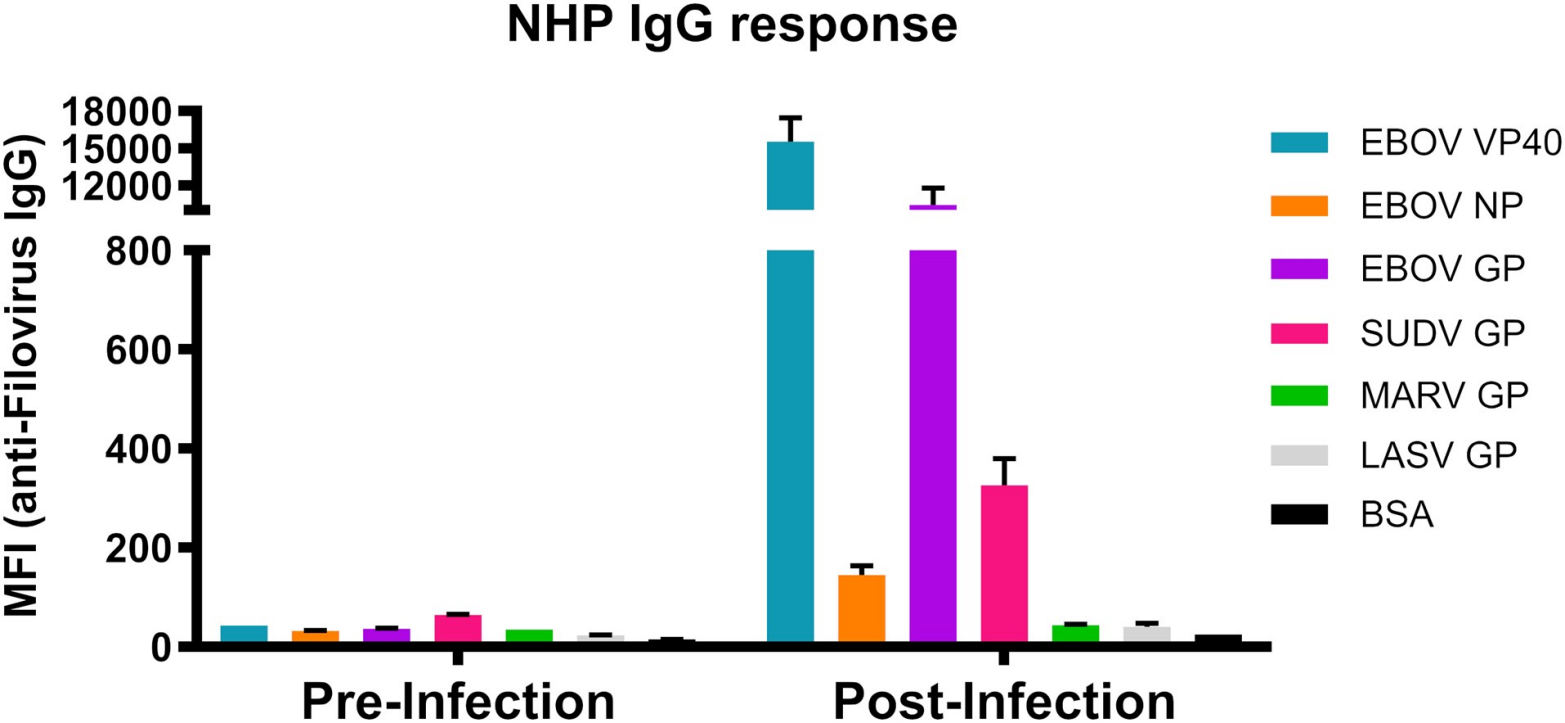
Detection of IgG response in an Ebola virus-challenged non-human primate using a custom-made microsphere immunoassay (MIA). Serum was collected from a single rhesus macaque prior to and 35 days after infection with Ebola virus. A multiplex MIA was conducted using recombinantly expressed antigens, as noted in the color key, coupled to beads using a serum sample diluted 1:1,000.

In this study, the primary goal was to establish a robust serological assay to test for filovirus antigen-reactive immunoglobulins using dog serum samples collected within a roughly 40 mile radius around Monrovia in Montserrado County, Liberia (Fig 1). Before deploying the assay to Liberia, reactivity and specificity was confirmed for all six viral antigens by testing serum samples from a rhesus macaque that survived infection with 1000 pfu of EBOV (Kikwit strain). The assay background was low in the pre-infected sample while the post-infection sample displayed elevated IgG titers to EBOV antigens GP, VP40 and NP as expected (Fig 2). In contrast, there was no increase in MARV GP and only minimal increase in SUDV GP antigen reactivity, demonstrating the specificity of the assay. Interestingly, the serum reactivity for VP40 post-infection with EBOV was higher than that against GP. This phenomenon may be related to the low amount of glycoprotein compared to VP40 present on each virion [16]. This observation corroborates previous studies which indicate that EBOV GP is not as immunogenic as VP40 [17]. Overall, the assay contained minimal background and displayed EBOV specificity.

**Fig 3:**
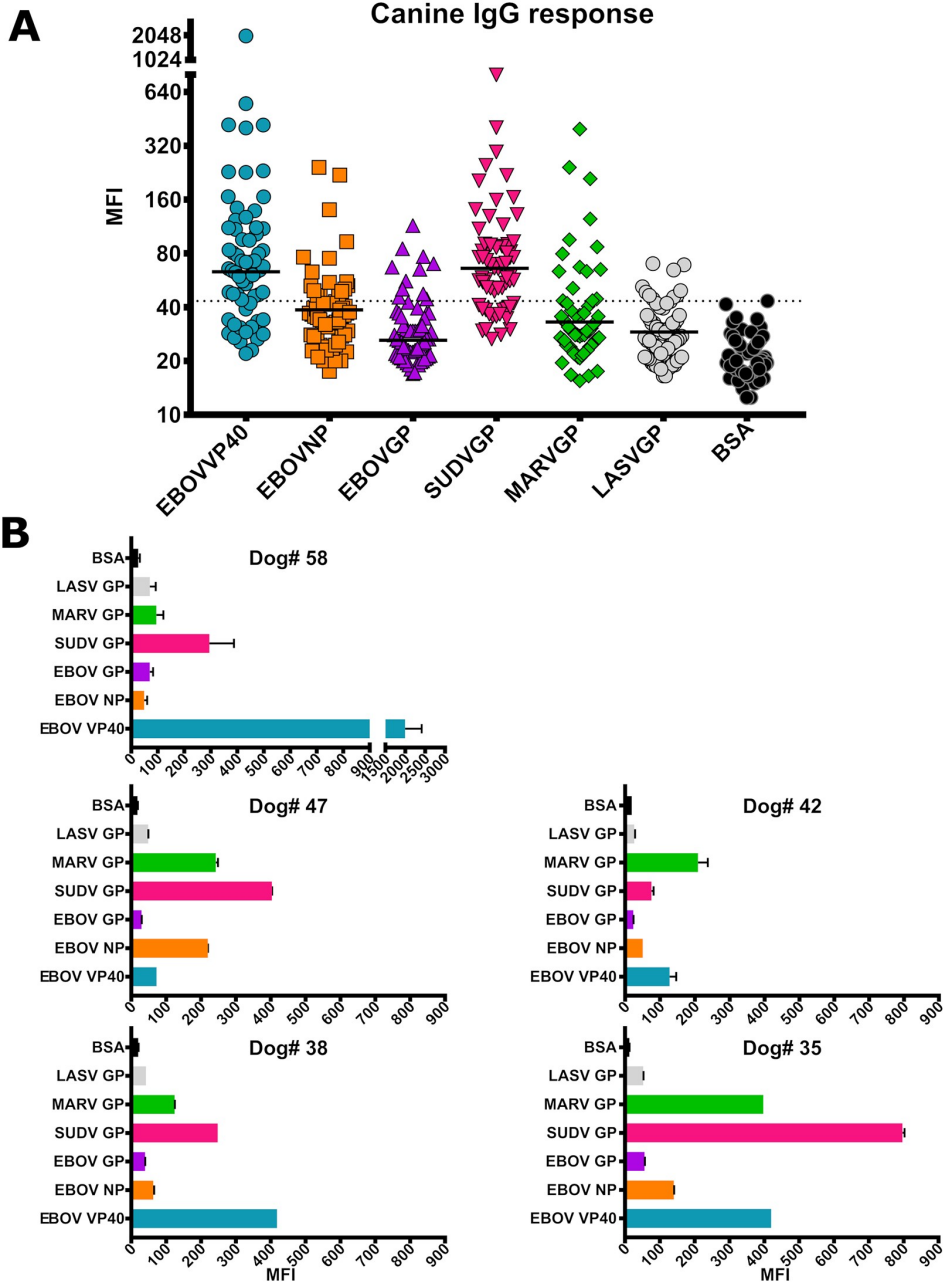
IgG responses to multiple recombinant filovirus and LASV GP antigens in dogs. (A) Serum samples from 64 dogs were diluted 1:200 and tested for Ebola and Lassa virus antigen-specific IgG responses using the MIA. Cut-offs were generated by determining the mean of 1/3^rd^ (22) serum samples showing the lowest MFI values plus three standard deviations (represented by the horizontal bars). If the cut-off value fell below the readout of the BSA control beads, the highest BSA-bead reading of 45 MFI was used (represented by the dotted line). (B) Serum samples, tested at 1:200 dilution, from five dogs demonstrating the highest IgG responses against multiple filovirus and Lassa virus antigens, expressed as MFI.

As the focus of the study was to determine if dogs could be used as sentinels of infectious diseases in Liberia, age appropriate dogs were selected from communities affected by the 2013–2016 EBOV outbreak. Detectable levels of IgG to several antigens offered evidence that the assay was working in the field (Fig 3). Specifically, 53.1% of the samples showed antibodies binding to EBOV-VP40, 26.6% reacted to EBOV-NP, 17.2% to EBOV-GP, 48.4% to SUDV-GP, and 23.4% to MARV-GP. In contrast, only 15.6% of samples reacted with LASV-GP (Fig 3A), showing a lower seroprevalence rate for this arenavirus known to be endemic in Liberia. Upon analyzing dogs that showed reactivity with more than one antigen, we found that dogs 58, 47, 42, 38, and 35 were the most highly reactive with multiple antigens (Fig 3B). Interestingly, one of these high titer dogs was from New Krew Town, one was from White Plains, and three were from Jerusalem community, respectively. This data may indicate hotspots of repetitive and/or early contact into Montserrado County, as the virus further spread into Monrovia and the community surrounding Redemption Hospital. Whether or not this indicates recent exposure in these communities is beyond the scope of this study but is important to consider nonetheless. Dogs 58 and 38 displayed high reactivity with EBOV-VP40 and SUDV-GP. Sample 42 appears highly positive for EBOV-VP40 and MARV-GP, whereas dogs 47 and 35 appear to display high reactivity with multiple antigens, having MARV and SUDV-GP in common. Collectively this data indicates that, in the field, canine serum samples appeared to show IgG reactivity with multiple filovirus antigens and almost no reactivity to LASV. Lack of LASV titers may be related to the sample origins’ proximity to the capital city of Monrovia. Recurrent studies of similar nature may help reveal if epidemiological information can be drawn from this type of surveillance.

While titers observed are overall relatively low, it is important to note that these samples were collected from animals 3–4 years after the height of the Ebola crisis, so it is not surprising that IgG titers may have waned. Samples from rural villages or dogs of a younger age may show different reactivity. Interestingly, the dogs also displayed the most reactivity to EBOV-VP40 and SUDV-GP similar to what was seen in the sample from an experimentally infected rhesus macaque (Fig 2). The overall relatively low EBOV-GP titers in the dogs may be due to the amount of GP antigen present on EBOV virions, or a sign that they may have been exposed to another, closely related virus. The low titers may also have resulted from animals being only exposed to infectious material, but not productively infected with any filovirus. This highlights a limitation of this study in that we were not able to assess viral status of these animals. Still, this data highlight the sensitivity of the multiplex assay, which may have impeded previous ELISA-based seroprevalence studies, such as those conducted in bats.

Earlier work from Gabon also reported seropositivity in dog sera [10] tested by ELISA with irradiated EBOV as the coating antigen. This study has experimentally-derived limitations. First, the origin and age of the animals from which samples were collected made it impossible to establish a negative baseline as no sera from confirmed unaffected dogs were available in the region and age-matched dog sera from other regions would not be a proper control due to different diet and health status. Therefore, our cutoff value for all analytes was based on the mean of 1/3^rd^ of dog serum samples showing the lowest MFI values plus three standard deviations. Furthermore, due to unavailability of confirmed EBOV positive canine sera we cannot assess the magnitude of infection these animals may have experienced. Despite these limitations, we identified 47 (73%) of 64 dog serum samples as potentially exposed to filoviruses.

**Fig 4:**
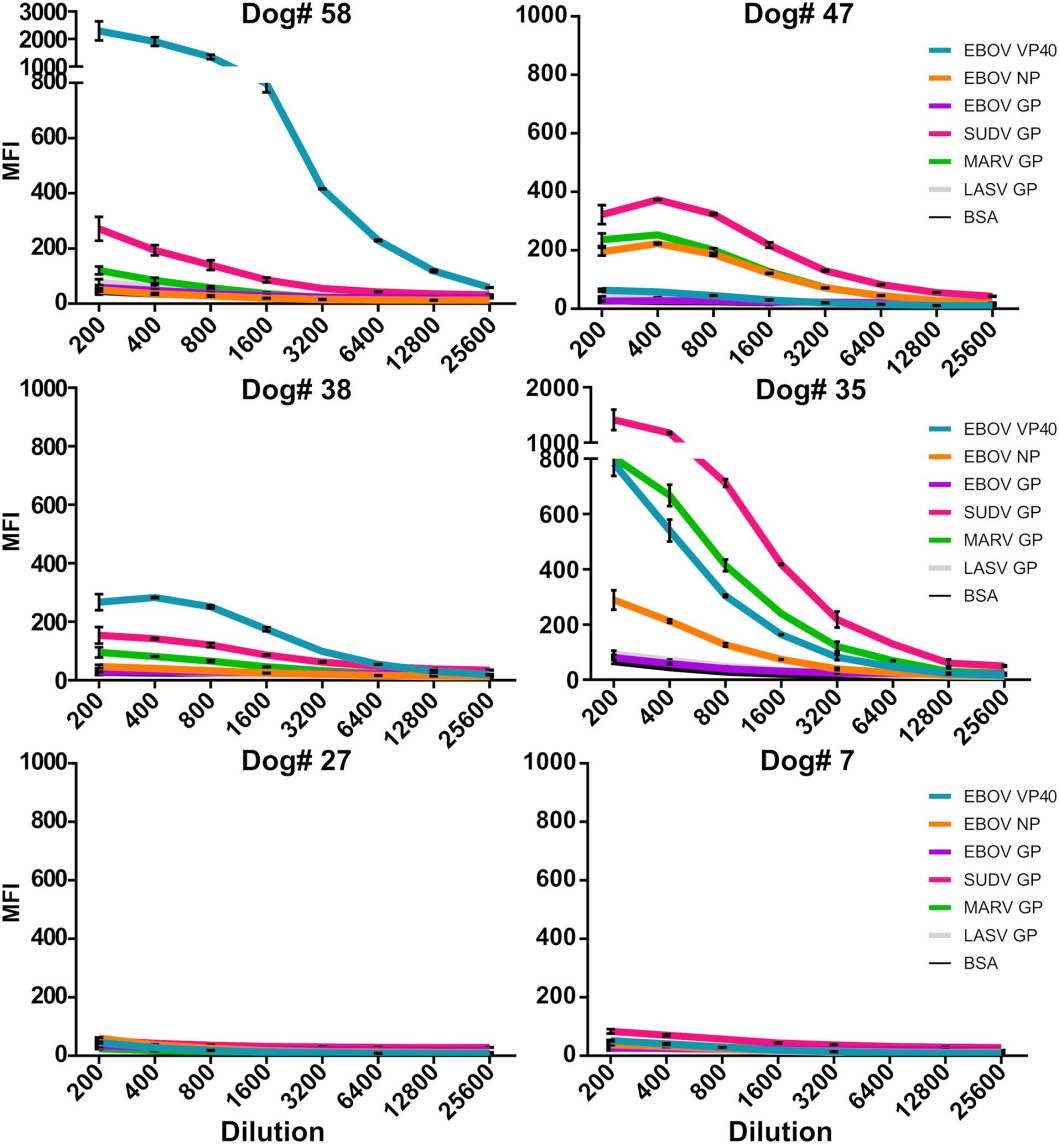
Serum IgG titration curves demonstrate robust or no reactivity to filovirus antigens. Four serum samples from dogs (numbers 58, 47, 38, 35) with high levels of reactivity to filovirus antigens and two dogs (numbers 27, 7) with low levels of reactivity were diluted two-fold from 1:200 to 1:25,600 for multiplex MIA analysis. BSA was used as a negative control.

The most common antigen reactivities observed were EBOV-VP40, SUDV-GP, and MARV-GP. Reactivity against SUDV-GP was generally higher than either EBOV, or MARV-GP. Considering that EBOV and MARV GP are rather distinct from SUDV GP among filoviruses [18], this finding could warrant further investigation. Since no SUDV cases have been reported in Liberia, this could also be due to the presence of related, yet unknown viruses (such as the recently reported Bombali virus [19]). Several dogs with apparent high IgG titers to one antigen proved to have high titers to multiple, but not all, antigens. The non-filovirus antigen reactivity remained relatively low for all dogs. To determine the robustness and specificity of the IgG reactivity, serial dilutions were conducted on several dog sera that appeared to have high titers to the recombinant filovirus antigens (Dogs #58, 47, 38, 35) as well as on samples from two dogs with no apparent IgG reactivity (Dogs #27 & 7). As expected, the reactivity (shown as MFI) reduces proportionally to the increase in dilution factor in the strongly IgG positive dogs whereas no change is observed in the dilutions from dogs appearing to be negative for filovirus antigen-specific IgG (Fig 4). This dose-dependent response further supports the multiple-reactivity data. Collectively, these phenomena may be an indicator of exposure to one or more filovirus particles acquired by canines from the environment.

The results obtained using the multiplex filovirus assay were also analyzed specific to different communities by determining the numbers of dogs in each town that were positive for IgG binding to at least one filovirus antigen. Interestingly, towns such as Blaton, White Plains, Dixville, New Kru Town, and Seonkpa had detectable IgG to the filovirus antigens in 100% of the samples tested (Table 1). White Plains, Dixville, and New Kru Town had the highest percentage of multi-positive dogs: 80%, 75%, and 80%, respectively. We recognize that some towns are less represented than others in this study and further targeted sampling of other townships would benefit future studies. We also observed a slightly higher proportion of females (78.7%) to males (62.1%), which displayed filovirus reactivity to one or more antigens. Thus, much information about the prevalence and impact of filovirus antigens in the environment can be gathered with this assay. Collectively these data reveal that the specificity and robustness of the developed MIA allow an efficient analysis of serum samples directly in endemic regions of Africa.

**Table 1:** Distribution of filovirus seropositive dogs based on town sites where the dogs resided.

*Town (n)*	*n (%) filovirus single antigen positive	*n (%) filovirus multi- antigen positive	*n (%) Males	*n (%) Females
***Yamah (22)***	10 (45)	3 (13.6)	5 (41.6)	4 (40)
***Blaton (4)***	4 (100)	1 (25)	1 (100)	3 (100)
***Korto*** *(6)*	4 (67)	3 (50)	1 (0)	4 (80)
***New Jerusalem*** *(11)*	8 (73)	8 (73)	2 (33.3)	5 (100)
***White Plains*** *(5)*	5 (100)	4 (80)	1 (100)	4 (100)
***Dixville*** *(8)*	8 (100)	6 (75)	5 (100)	3 (100)
***New Kru*** *(5)*	5 (100)	4 (80)	2 (100)	3 (100)
***Seonkpa*** *(3)*	3 (100)	1 (33)	-	-
***Total (64)***	**47 (73.4)**	**30 (46.8)**	**18 (62.1)**	**26 (78.7)**

*Positive serum samples defined as serum samples above the cutoff and multi-antigen positive defined as serum samples positive for two or more antigens.

The implications of animals testing positive for filovirus antigens other than EBOV surrounding Monrovia are perplexing. It has peaked our interest that the assay was specific in a controlled setting (Fig 1) yet broad reactivity was observed with greater than expected frequency in the field. Since this is four years post-outbreak, it is difficult to speculate what may be causing broad reactivity. We suspect that humoral responses to antigen acquired through a contaminated meal, instead of virus being injected Intramuscularly as in animal disease models, may be one explanation for these variances. Future work may include IgA titer analysis as well to elucidate mucosal responses. Overall, this assay should also be applied to places with recent or ongoing EBOV outbreaks so that we may be able to determine how broad the reactivity profile is in such settings. Of importance would be to determine the utility of this method on human sera as well.

In conclusion, we observed high numbers of dogs positive for filovirus-specific antibodies throughout Liberian communities in Montserrado County. Our data indicate that the multiplex MIA is specific and robust at detecting filovirus-reactive IgG in dogs. Further, these data support the feasibility of MIA to detect anti-filovirus IgG in a field setting, and suggest that dogs may serve as accidental EBOV sentinels. Previous studies have used ELISAs, which are limited not only in sensitivity but also by the number of antigens that can be tested for in a single assay. In contrast, the multiplexed MIA allows for rapid detection of immunoglobulins against a variety of antigens in a single run. This has the advantage of reducing the sample volume and assay materials, thereby easing transportation while allowing deployment to the field. Collectively, these advantages allow this assay to be effectively executed in resource- and infrastructure-poor regions of the world. We hope these data can provide a foundation for further filovirus-specific sero-epidemiology research in domesticated and wild animals in West Africa.
